# Bifid Mandibular Condyle With Associated Temporomandibular Joint Ankylosis: A Rare Skeletal Abnormality

**DOI:** 10.7759/cureus.29624

**Published:** 2022-09-26

**Authors:** Connor D Michalski, Andrew Pollizzi, Deeksha Dhar, Laura L Hayes, Tushar Chandra

**Affiliations:** 1 Radiology, University of Central Florida College of Medicine, Orlando, USA; 2 Medicine, Government Medical College and Affiliated Hospitals, Jammu, IND; 3 Pediatric Radiology, Nemours Children's Hospital, Pensacola, USA; 4 Pediatric Radiology, Nemours Children's Hospital, Orlando, USA

**Keywords:** temporomandibular joint (tmj) disorders, temporomandibular joint imaging, mandibular imaging, congenital defects of mandible, temporomandibular joint ankylosis, mandibular anomalies, bifid mandibular condyle

## Abstract

Bifid mandibular condyle (BMC) is splitting the mandibular condyle into two separate articular surfaces. The etiology is poorly understood, but trauma and developmental issues are currently the most cited causes. Though most often asymptomatic, occasionally, this condition may cause the development of jaw pain, clicking, and restriction of motion. We present a rare case of a patient who developed unilateral ankylosis of the temporomandibular joint (TMJ) secondary to BMC in the absence of trauma or infection. The ankylosis developed due to abnormal biomechanical forces and degenerative arthritis secondary to the abnormal articulation of the TMJ caused due to BMC. CT imaging is the best modality to evaluate the bony anatomy of the TMJ. It is essential to consider BMC as a cause of TMJ pathology, as management is primarily surgical in nature.

## Introduction

Bifid mandibular condyle (BMC) is a rare skeletal abnormality that involves the separation of the mandibular articular condyle into two articular surfaces. These surfaces divide either anteroposteriorly or mediolaterally [1-3]. Previous research suggests that mandibular trauma promotes the development of the anteroposterior form. In contrast, developmental anomalies account for the mediolateral form of BMCs, but this is not an all-or-nothing rule [4,5]. BMCs are often found unilaterally and do not cause symptoms [5]. We present a rare case of symptomatic, unilateral BMC with associated ankylosis of the temporomandibular joint (TMJ) in the absence of a history of trauma, surgery, or infection of the jaw.

## Case presentation

A nine-year-old boy presented with a six-month history of progressive jaw deviation and difficulty opening his mouth, with a sensation of the left side of his jaw feeling “stuck.” There was no reported recent or remote history of trauma, surgery, or jaw infection. In addition, the patient had no documented history of metabolic or genetic skeletal abnormalities and was otherwise healthy.

A significant leftward deviation of the mandible was noted on physical examination relative to the maxilla and skull base. A significant overbite was also appreciated. The facial movement was normal, but the jaw opening was limited. No facial masses or cervical lymphadenopathy were noted. A CT of the maxillofacial region with and without contrast was obtained to further evaluate this condition. The contrast was administered to confidently rule out infection, despite the absence of clinical signs such as fever, erythema, and edema.

**Figure 1:**
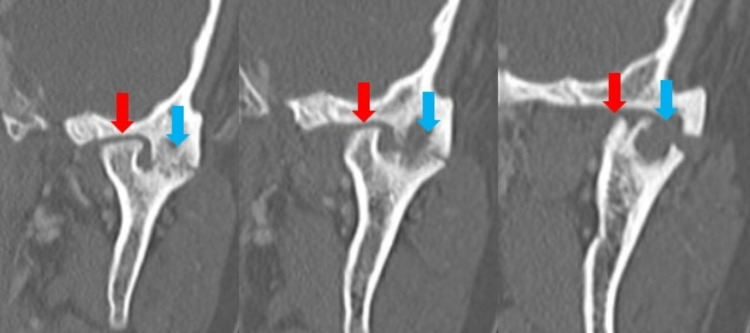
CT images of the left TMJ in coronal view. Flattened medial articulation (red arrow) and lateral articulation with osseous bridging and erosive changes (blue arrow) of bifid mandibular condyle. TMJ: Temporomandibular joint.

**Figure 2:**
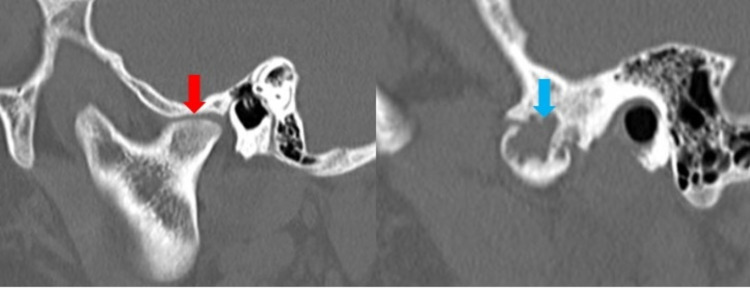
(A) CT image of medial articulation of BMC of the left TMJ in sagittal view; (B) CT image of lateral articulation of BMC of the left TMJ in sagittal view. a) Flattened articulation shown (red arrow) b) erosive joint changes (blue arrow). BMC: Bifid mandibular condyle; TMJ: Temporomandibular joint.

CT imaging demonstrated an abnormal left TMJ with two sites of articulation oriented medially and laterally. The medial component of the condyle appeared to articulate with the temporal process of the zygomatic bone with an abnormally flattened appearance. The medial articulation showed no joint narrowing, erosion, or lytic lesions (Figures 1-2A).

**Figure 3:**
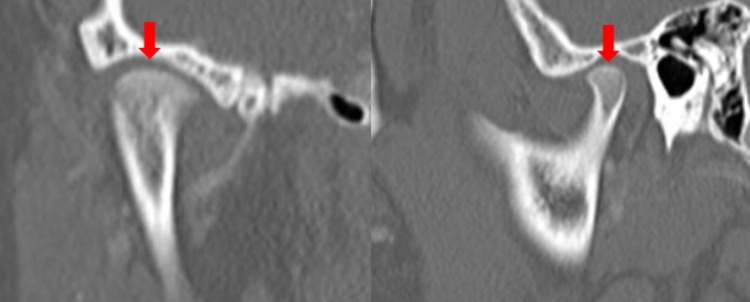
CT images of normal right TMJ shown in (A) coronal view and (b) sagittal view. Normal curved articulation (arrows).

**Figure 4:**
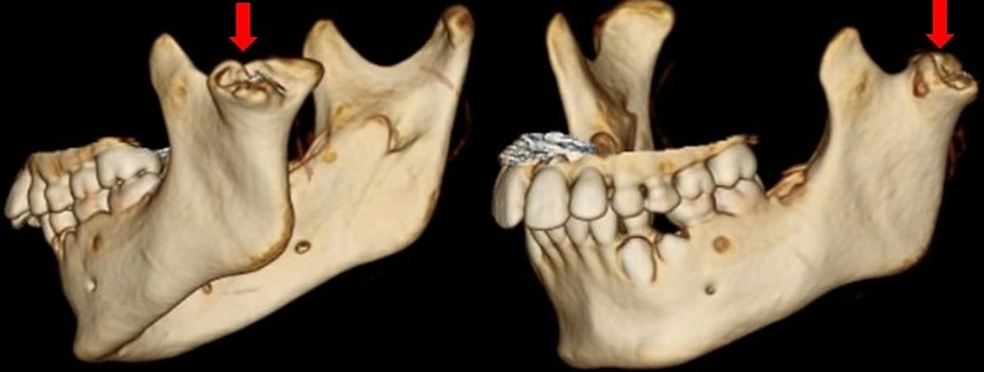
3D reconstructions with surface rendering. Left BMC shown in red arrows. BMC: Bifid mandibular condyle.

**Figure 5:**
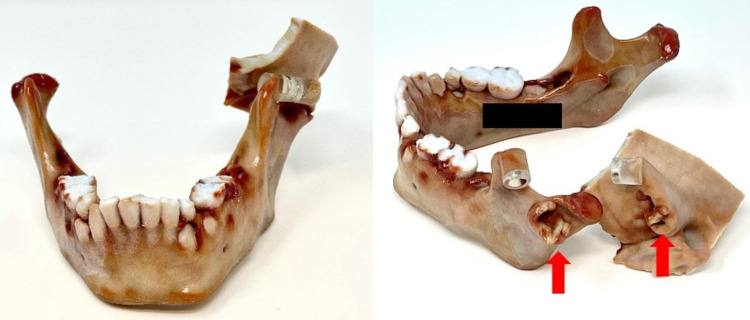
3D printed model of BMC of our patient. Erosions of the lateral articulation of BMC and temporal bone can be seen on both surfaces composing the TMJ (red arrows). BMC: Bifid mandibular condyle; TMJ: Temporomandibular joint.

The lateral articulation appeared to have an osseous bridge extending between the lateral mandibular ramus and the lateral process of the temporal fossa. This “joint” space was narrow, with significant erosive changes in both opposing surfaces of the articulation. There was no evidence of periosteal reaction, involucrum, or additional lesions. No soft tissue changes, fat stranding, edema, or fluid collections were appreciated (Figures 1-2B). The right TMJ was normal in appearance (Figure 3). 3D reconstructions and a 3D-printed model of the mandible demonstrating BMC can be seen in Figures 4-5, respectively.

## Discussion

BMC is a rare anatomic abnormality with an estimated prevalence of 0.018%-1.82% [6,7]. Some authors speculate that the condition may be more common than previously thought as it is usually asymptomatic and usually found incidentally [8]. The etiology of BMC is controversial, but several possible causes have been proposed, including etiologies of traumatic, infectious, vascular, developmental, teratogenic, or endocrine origins [3]. Several studies have shown that unilateral BMC is much more common than bilateral BMC [5]. BMC is often asymptomatic, but ankylosis of the TMJ may develop and present with jaw clicking, pain, and limited jaw opening. In previous cases of BMC with ankylosis, trauma and infection were proposed to be the causes [9].

BMC is often found incidentally on panoramic dental radiographs or head and neck imaging [2,10]. CT imaging is the best tool to evaluate the TMJ’s bony structures and articulations. MRI is useful for evaluating the soft tissues, cartilage, and disc within the joint space [10]. Ultrasound and shear wave elastography have also shown some benefits in evaluating disc and other fibrocartilaginous structures [11,12]. The imaging findings in our patient include osseous bridging and lytic changes to both surfaces of articulation in the lateral bifid condyle, which are most consistent with ankylosis of the TMJ joint. A report by Rehman TA et al. [9] presents similar images of the TMJ with ankylosis. Differential diagnoses for these imaging findings include focal TMJ osteomyelitis, osteosarcoma, and osteochondroma. Focal TMJ osteomyelitis is exceptionally rare and would be unlikely in the absence of systemic symptoms and local pain. There is also no radiographic evidence of involucrum, which may be seen with osteomyelitis [13]. Osteosarcoma of the jaw is also rare, occurs more commonly in older individuals, and would most likely demonstrate periosteal elevation with a “sunburst” appearance [14], which this patient did not have. It would also be exceptionally rare for an exophytic lesion, such as osteochondroma, to involve both opposing surfaces of an articulation.

There have only been about 50 reported cases of BMC with associated ankylosis [9,15-19]. All of these studies implicate that the cause of ankylosis was either prior trauma or infection. This was not the case in our patient, as these factors were absent from the patient’s history. This patient’s BMC was a developmental defect, as there was no evident precipitating cause. We propose that the TMJ ankylosis in our patient was caused by the anatomic variation of the BMC and the redistribution of biomechanical forces, which resulted in severe arthritis and degenerative changes to the joint with reactive ankylosis.

Management of mandibular ankylosis is typically surgical in nature. Prior successful treatment has been achieved with condylectomy, bilateral coronectomy, and genioplasty to correct chin deviation [20]. Mild cases may be managed with muscle relaxants, non-steroidal anti-inflammatory drugs (NSAIDs), and physiotherapy [5]. Due to the severity of the symptoms, our patient was referred to an oromaxillofacial surgeon for surgical management. Unfortunately, this patient did not appear for follow-ups.

## Conclusions

In our case, BMC presented with rapidly progressive symptoms and ankylosis in the absence of trauma or infection. Thus, it is essential to consider mechanical changes as a cause of ankylosis in patients with BMC. Though BMC is rare and continues to be poorly understood, it should be considered when there is a pathology of the TMJ. CT imaging should be obtained to evaluate the bony anatomy of the TMJ if pathology is suspected. Regarding management, surgical evaluation should be obtained for patients with symptomatic BMC. There are no clear guidelines to direct either surgical or medical management. However, a decision should be made based on the severity of the patient’s symptoms and their impact on quality of life.
